# Comparative study of the effectiveness of the surgical technique with and without preservation of the conchal cartilage in otoplasty through the measure of the cephalo-auricular and scapho-conchal angles

**DOI:** 10.1016/j.bjorl.2022.12.002

**Published:** 2022-12-14

**Authors:** Caio Marcio Correia Soares, Flávia David João De Masi Nassif, Daniela Dranka, Renata Vecentin Becker, Johann Melcherts Hurtado, Renato da Silva Freitas

**Affiliations:** a Universidade Federal do Paraná (UFPR), Departamento de Otorrinolaringologia, Curitiba, PR, Brazil; b Hospital IPO (Instituto Paranaense de Otorrinolaringologia), Curitiba, PR, Brazil; c Hospital do Trabalhador, Curitiba, PR, Brazil; d Núcleo de Ensino e Pesquisa (NEP) do Hospital IPO (Instituto Paranaense de Otorrinolaringologia), Curitiba, PR, Brazil; e Universidade Federal do Paraná (UFPR), Hospital de Clínicas, Curitiba, PR, Brazil

**Keywords:** Otoplasty, Prominent ears, Measurement

## Abstract

**Objectives:**

Prominent ear abnormalities affect 5% of the population. A prospective, double-blind study of patients who underwent otoplasty procedures to correct these abnormalities was conducted to compare two surgical techniques. They diverge by the preservation or not of conchal cartilage.

**Methods:**

The two techniques were compared by measuring the cephalo-auricular and scapho-conchal angles. Measurements were performed in pre and 6-months post-operative periods using alginate molding. Twenty patients were randomly assigned to two groups (with and without cartilage preservation) with 10 participants each. Student’s *t*-test, Covariance Analysis Model (ANCOVA), and non-parametric Mann–Whitney were used in the statistical analyses.

**Results:**

A significant reduction in the average of the cephalo-auricular and scapho-conchal angles was observed in both surgical procedures (*p* < 0.001). However, no significant difference was found between them (*p* = 0.887).

**Conclusion:**

The two techniques analyzed in this study fulfilled their objectives. Therefore, further comparative studies are needed to confirm the superiority of one over the other.

**Level I:**

Evidence obtained from at least one properly designed randomized controlled trial.

## Highlights

• Protruding ear can causes low self-esteem, leading patients to undergone otoplasty.
• Otoplasty with or without conchal preservation significantly reduce the deformity.
• Careful analysis of the auricular deformity to choose the best surgical technique.
• Eclectic technique with conchal remaining suture is effective for ear correction.
• No significant difference between otoplasty with and without conchal preservation.

## Introduction

Prominent ear occurs in 5% of population, in both gender and may be uni or bilateral.^1^ This abnormality includes underdevelopment of the antihelix, overdevelopment of the deep concha, increases cephalo-auricular and scapho-conchal angles, and protrusion of the lobule. Protruding ear frequently causes low self-esteem and bullying, taking the patients to undergone otoplasty.

Ely (1881) described the first technique to treat protruding ear that consisted in skin and cartilage incision.^2^ Lucket (1910) recognized the antihelix importance to this malformation.^3^, ^4^ Mustardé proposed multiples sutures on the posterior face of ear cartilage, to create a natural smooth antihelix.^5^ Furnas contributed with correction of concha repositioning making sutures between concha and mastoid periosteum. Others described many variations of these principles to achieve natural look and persistent results.^6^ Stucker (1977) associated controlled conchal resection to Mustardé stitches, adding a new surgical option with few complications, and low tax of recurrence.^7^ All these techniques may be divided in two groups: with or without concha cartilage removal.^8^ Resection of concha aims to eliminate the cartilage memory and reduces the tension between the sutures and consequently recidive. However, it can lead to lose of ear natural contour.^9^

The objective of this study is to compare two techniques in the treatment of prominent ear, focusing at concha resection, to better understand the real effect of this step in otoplasty.

## Methods

A prospectively, double-blinded, and randomized evaluation of twenty patients submitted to bilateral otoplasty, from October 2014 to November 2016. Randomization was conducted by alternatively assigning patients to each treatment, previously determining that the first hospitalized patient demanding the surgical intervention would be assigned to otoplasty with preservation of the conchal cartilage, the second to intervention without preservation of the conchal cartilage and continuing in this way following the order in which patients were hospitalized. Thus, subjects with even or odds numbers were assigned to otoplasty treatments with or without preservation of the conchal cartilage, respectively, until 20 patients were attended (two groups with ten individuals each). All the patients underwent an alginate ear molding before the surgery and six months later. The Eclectic Technique, described by Maniglia, was chosen to all cases.^10^ This study was approved to the Ethics and Research Committee — CAE 37704114.7.0000.5529.

### Preoperative approach

The complaints and expectations of patients and family members were widely discussed. Anatomical changes were observed and evaluated comparing the two ears: anti-helix non-formation, the concha overdevelopment, the anti-helix upper crux concavity, the earlobe protrusion, the pavilion topography and the auricular cartilage thickness. Consent term was applied and signed to all patients before surgery, as well as standardized photographic documentation.

### Surgical technique

Patients underwent otoplasty using the technique described by Maniglia et al.,^10^ with some modifications by the senior author, who conduct all the surgical interventions on both ears of every patient. Local anesthesia with sedation was preferable after 12 years of age and intravenous antibiotic prophylaxis with first generation cephalosporin was performed one hour before beginning the surgical procedure. Xylocaine® 2% with adrenaline 1:100,000 was used for local anesthesia. Infiltration started through the retroauricular groove superficially and through the deepest region of mastoid. Subsequently, the convex face of the ear via hydro dissection. In the anterior region, the concha was infiltrated facilitating the detachment of the skin from the cartilage. Even in cases with general anesthesia, local infiltration was done to improve hemostasis (Supplementary material_Video).

**Fig. 1:**
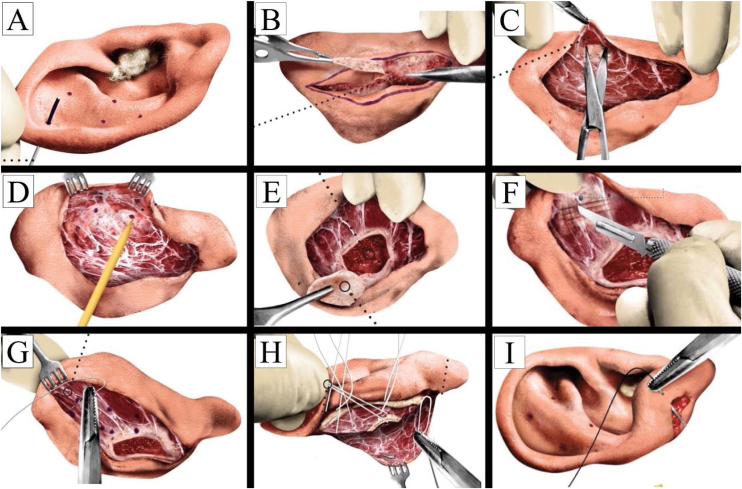
Surgical steps of otoplasty. (A). Mustardé tattoo points; (B) retroauricular incision; (C) dissection; (D) identification of tattoo; (E). Concha resection; (F) cartilage incision; (G) Mustardé sutures; (H) furnas sutures; (I) lobule repair.

A retroauricular skin ellipse was drawn about eight to ten millimeters medially to the edge of the helix and anteriorly to the retroauricular groove. The excision should not be exaggerated so that the suture can be performed without tension. Here, it was important to check the position of the lobe, as the incision should be extended through the earlobe (Fig. 1).

The new anti-helix is simulated with a digital maneuver, and then the markers were tattooed with a straight needle and gentian violet trough the cartilage. At least three or four points are marked to do the sutures, as described by Mustardé.

The incision and removal of retroauricular skin began inferiorly so the bleeding will not affect the upper incision view. The dissection plane was between the subcutaneous tissue and the perichondrium. With an iris scissors or a scalpel, the pre-marked skin was removed. Careful hemostasis was performed. At this point it was important to highlight the preservation of the perichondrium.

A lateral skin flap was made wide enough to visualize the demarcated site for Mustardé sutures. This procedure aimed to avoid threads extrusions in the postoperative period. The posterior ligaments and extrinsic muscles of the ear were sectioned, with the mastoid periosteum as the medial limit. After this step, the ear was loose, which allowed to correct, through sutures, the topography of the pavilion in a side view, as well as its retropositioning.

The removal of conchal cartilage excess were established by two points: an upper and a lower one. It was important to make sure that the cartilage to be removed should not coincide with the markings of the Mustardé sutures. The extension in the smallest axis of the ellipse was proportional to the deformity to be corrected. A 15-blade scalpel was utilized to incise the cartilage, and with a freer detacher, the conchal cartilage can be easily dissected, leaving the perichondrium attached to the skin, without impairing its circulation and consequent skin vitality.

The new anti-helix area was weakened through incomplete longitudinal and transverse incisions in the cartilage. The depth of the incision will depend on the thickness of the cartilage.

Mustardé sutures were positioned and repaired using 4.0 colorless nylon® thread. U-shaped sutures were performed as previously marked, sliding the needle between the cartilage and the anterior skin of the auricular pavilion. Three or four sutures were performed, according to cartilaginous thickness: in the thinnest cartilages, three sutures were sufficient; in the thick ones, up to four master sutures were performed.

The fixation of the remaining concha in the mastoid region allowed to reposition the auricular pavilion, providing its retropositioning with superior rotation. Four sutures with Vicryl® 3.0 thread was performed. The first suture was positioned approximately five millimeters from the upper vertex of the removed ellipse, medially. The second suture, also at 5-mm, at the lower vertex. Therefore, the third suture was placed at the upper vertex, laterally, and the fourth, at the same distance from the lower vertex (Fig. 1). For a natural result the last two lateral sutures are fixed as posteriorly as possible (subcutaneous layer), parallel to the skin incision. In the surgical technique where the conchal cartilage was preserved, the same technique of suture sites was used, however without removing the excess conchal cartilage.

The Mustardé sutures were carefully finished, with gradual and progressive tightening. Cartilage folds should be subtle and rounded. The distance between the helix and the mastoid around 13–15 mm was respected. A slight two-millimeter overcorrection is appropriate.

The last structure to be corrected was the lobule. Black nylon® 4.0 thread was used. The position of the lobe should coincide with the auricular pavilion plane. This uneven suture should be performed in the most prominent region of the lobe, simulating its retropositioning. The skin was sutured with simple, separate stitches, without tension, to avoid the appearance of hypertrophic scars, keloids and/or skin infections.

The distances between the lateral edge of the helix and the surface of the mastoid were measured in its upper, middle and lower thirds before dressing. A cotton molded wrapped in antibiotic ointment was placed in the concha region, reducing the possibility of dead space and hematoma. A 12-cm wide crepe bandage should be attached around the head, taking care that there is not too much tight.

### Post-operative guidelines

Hospital discharge occurred few hours after surgery. The prescription included antibiotics for seven days and common painkillers. A compressive elastic band was used for 24 h for 30 days and, for the next 30 days, only during the night. They were also instructed to avoid intense physical activity and sun exposure for 60 days.

The first return occurred in 72 h, when the compressive dressing was removed. A second return was between 10 and 12 days to remove retroauricular sutures. The photographic documentation was made in the first, third and sixth months after surgery.

### Pre and postoperative measures

Ear molding was performed using alginate (Dencrigel® brand) preoperatively and six months after surgery. The first cut is made transversely, in the middle of the cephalocaudal length of the ear. The second cut is made between the first cut and the upper end of the ear, at an equal distance.

The cephalo-auricular angle measure used the first cut. It was defined as the intersection between the lateral portion of the mastoid with a line passing between the tragus and the middle of the helix. The scapho-conchal angle is obtained in the second cut, with the same structures in a posterior view of the ear.

The molds were photographed using a Sony Cyber-Shot® 7.2 megapixel camera with Carl Zeiss Vario Tessar® 2.8–5.8/5.35‒21 lens, 4. The photos were sent for an external examiner to measure the scapho-conchal and cephalo-auricular angles, using the computer program Onde Rulers using the Protactor Ruler function.

### Statistical analysis

The results of the study variables were described by means, standard deviations, medians, minimum and maximum values. To compare the two surgical techniques, the Student’s *t* test for independent samples was used. This comparison in relation to the evaluation after six months of surgery was made considering the covariance analysis model (ANCOVA) adjusted for the pre-operative evaluation. For comparisons between surgical techniques in relation to the reduction percentage after six months, the Mann–Whitney non-parametric test was used. Hotelling’s T2 statistic was used to compare the vectors of the two variables in the study. Kolmogorov–Smirnov test assessed the condition of normality of the variables. Values of *p* < 0.05 indicated statistical significance. The data were analyzed using the IBM SPSS Statistics v.20 computer program. It was assumed that the observation units are independent regarding the evaluation of the variables of interest.

## Results

**Fig. 2:**
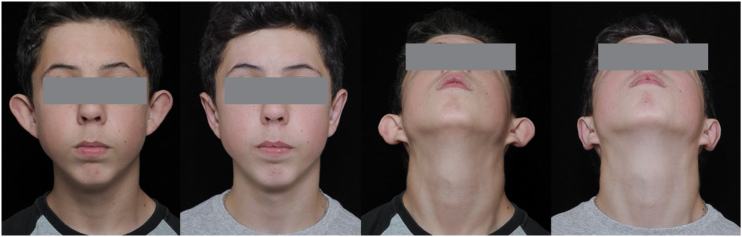
Pre- and post-operative picture of patient with concha resection.

**Fig. 3:**
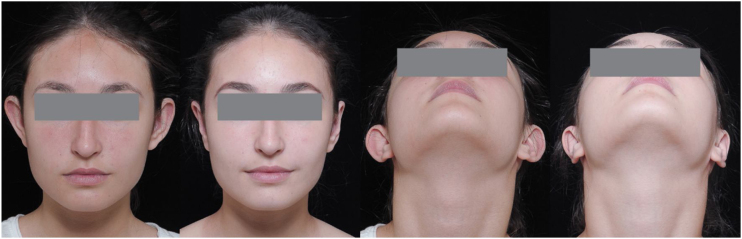
Pre- and post-operative picture of patient without concha resection.

Twenty patients were submitted to bilateral otoplasty. All the patients underwent an alginate ear molding before the surgery and six months later. One group (10 patients) were submitted to the technique with partial removal of the concha (Fig. 2) and the other group (10 patients) with preservation of the concha cartilage (Fig. 3), randomly. The cephalo-auricular and scapho-conchal angle were measured in all patients according to de predetermined period.

In the preoperative period, the cephalo-auricular angle did not present statistical difference between two groups, with a mean of 41.5 in patient when it was performed conchal resection, and 43.0 without resection. After surgery, the angle was 30.0 and 29.0 respectively, also without any significant difference. When it was evaluated the angles in two times, pre- and post-operative period, for the technique without conchal removal, a significant difference was found between the measurements before and after six months (*p* < 0.001), so as for conchal removal technique. It is demonstrated in Table 1 that both surgical techniques have significant reduction in the cephalo-auricular angle.

**Table 1:** Evaluation of the cephalo-auricular angle according to the type of technique.

Cephalo-auricular angle	Resection	n	Average	Median	Minimum	Maximum	Δ	*p*
Pre-op	+	20	42.9	41.5	30.0	56.0	8.1	0.506^a^
	–	20	44.8	43.0	28.0	61.0	10.2	
Post-op	+	20	29.9	30.0	23.0	39.0	4.4	0.505^b^
	–	20	29.3	29.0	20.0	40.0	6.5	
Absolute reduction	+	20	13.0	11.5	2.0	25.0	7.6	0.350 a
	–	20	15.5	13.0	0.0	32.0	9.3	
%	+	20	28.5%	27.7%	5.4%	51.1%	13.5%	0.301^c^
	–	20	32.8%	34.8%	0.0%	59.3%	15.8%	

a Student’s *t*-test, *p* < 0.05.

b ANCOVA.

c Mann–Whitney *U* test.

Table 2 presented descriptive statistics of the scapho-conchal angle evaluations according to the technique. The *p*-values of the statistical tests are also shown. In the preoperative period, the scapho-conchal angle in the group with and without removal of the auricular concha did not show statistical difference, with a mean of 128.5 and 126.5, respectively. After surgery, the angle was 100.0 and 97.0 respectively, also with no significant difference.

**Table 2:** Evaluation of the scaphoconchal angle according to type of technique.

Scaphoconchal angle	Resection	n	Average	Median	Minimum	Maximum	Δ	*p*
Pre-op	+	20	127.6	128.5	91.0	169.0	25.1	0.962^a^
	–	20	127.3	126.5	99.0	162.0	21.4	
Post-op	+	20	98.9	100.0	70.0	126.0	13.2	0.504^b^
	–	20	96.4	97.0	79.0	118.0	11.7	
Absolute reduction	+	20	28.7	22.5	−14.0	66.0	25.3	0.748^a^
	–	20	30.9	31.0	−1.0	60.0	15.8	
%	+	20	20.1%	20.6%	−14.4%	41.1%	16.6%	0.738^c^
	–	20	23.2%	25.4%	−1.0%	38.0%	9.5%	

a Student’s *t*-test, *p* < 0.05.

b ANCOVA.

c Mann–Whitney *U* test.

For pre-surgical evaluation there was no significant difference between the two techniques considering together the two variables (*p* = 0.803). Also, for the evaluation after six months, no significant difference was found between the two surgical techniques (otoplasty with conchal resection; and without resection (*p* = 0.816). When comparing the techniques as reductions in the two variables after six months, there was no significant difference (*p* = 0.601).

## Discussion

The complications of otoplasty are uncommon. Keloids, hypertrophic scars, infections and hematoma can occur. Converse, in 570 ears studied, reported 1.2% infection rate, 0.8% recent postoperative hematoma, 2.1% keloid formation in Caucasians and 11% in people of African descent.^11^ Milojevic, in 244 cases, had no infections or perichondritis, 0.8% post-surgical hematoma and only 0.4% retroauricular keloid formation.^12^ However, both authors published recurrence of deformity in 5.6% and 2.8% respectively. This complication is technical related, and most of surgeons work to avoid it.

For years, hundreds of techniques have been described to correct auricular deformities.^4^, ^10^, ^13^, ^14^, ^15^ The correction of prominent ears consists of choosing the most appropriate surgical technique according to the anatomical deformity. The results are influenced by the analysis of the deformity degree, as well as by the surgeon’s skills and experience, minimizing the possibility of post-operative complications.^16^ Articles and publications also mention otoplasty without incision, in which percutaneous sutures are performed to define the anti-helix, as described by Fritsch.^17^

There are two possibilities to correct the conchal hyperdevelopment, mastoid fixation and conchal resection. The conchal degree of development should be analyzed. The conchal-mastoid sutures described by Furnas are effective in mild to moderate deformities, where the conchal height reaches up to 2.5 cm.^18^ It recommends four to five horizontal sutures, joining the concha posteriorly to the mastoid face with Mersilene® 4–0. The complement with the Mustardé master sutures should have as a parameter a pleasant relationship with the scapho-conchal angle around 90°.

Excisions with partial conchal removals, addressed by retroauricular access, tend to have a guarantee of non-recurrence, with more predictable results of the distance between the edge of the helix and the retroauricular region.

The cartilage preservation technique is more conservative when compared to cartilage resection. They are ideal for patients with flexible cartilage and minimal and/or moderate deformities. Complications, such as post-surgical hematomas, are rare in these cases, in addition to decreasing management and surgical time. A greater example would be the master sutures discussed classically by Mustarde.^19^ However, there are reports of higher rates of recurrence, requiring surgical revisions more frequently.^9^

The resection techniques aim to eliminate the cartilage memory to modify the shape of the ears. Therefore, it is described as a technique to avoid recurrence. They are more suitable for patients who have rigid cartilage structure with increased thickness. However, they may lead to important asymmetries.^9^

The combination of procedures could be one of the reasons that contribute to the maintenance of long-term stable distances between the edge of the helix and the retroauricular region, as in the eclectic technique, described by Maniglia, allowing a more natural and lasting result.^10^ Maniglia et al.^10^ contributed to several publications reporting their technique and standardization. The main advantage of this technique includes total control of mastoid anchorage and low implantation correction, with the possibility of retropositioning and superior rotation of the pavilion without anterior incisions in the auricular pavilion. There is also easy applicability, as well as a low rate of complications and protrusion recurrence. The learning curve is progressive. The surgical steps must be respected, because, if they are not effective, they can be repeated, not jeopardizing the steps already carried out, stimulating the aesthetic sense.

There are countless reasons to associate the concha and anti-helix correction. The eclectic technique removes the concha along the suture of the remaining cartilage in the mastoid region, preventing the narrowing or collapse of the external auditory canal.^20^ The interruption of conchal cartilage makes its transition with antihelix less evident. Associated with the Mustardé sutures, they recreate the ear with a more natural and elegant appearance. The cartilage removal can cause a projection decrease of the antihelix.^21^

There is no consensus in the literature on the superiority of one technique. Toplu et al.,^22^ in a study with 132 ears, stated that long-term otoplasty without cartilage removal and based on aesthetic results and patient satisfaction index, would be better compared to the technique with conchal removal. Kompatscher et al.^23^ also obtained similar results in a study with 281 patients. Panettiere et al.^24^ analyzed 104 ears and did not evidence long-term difference between the techniques. However, Bauer et al.^25^ advocated conchal resection, because despite many techniques discuss antihelical fold as the key component in correcting the prominent ear, in his experience the principal cause is the conchal hypertrophy having a benefit with chondrocutaneous resection.

Our study obtained a similar result as described by Panettiere, since no statistical significance was found between the two techniques six months after the operation. Observing the differences between the cephalo-auricular angle and the scapho-conchal angle, it was noted that both techniques make the ear aesthetically acceptable. For the cartilage preservation technique, a significant difference was found between the measurements before and after six months (*p* < 0.001). For the removal technique, there is also a significant difference between pre and post six months (*p* < 0.001). However, no significant difference was found between the techniques six months after the operation (*p* = 0.887). Our results come to confirm that both approaches may obtain adequate result until 6-months of follow-up. Studies with longer analysis may prove the security of these procedures in relation to recurrence.

Our study is based on a sample size including two groups of ten patients each, representing the surgical intervention of 40 ears, which outcomes were analyzed by proper statistical methods. Hence, even considering that analyzing a sample size larger than the one used in our study can bring new perspectives concerning our findings, our results remain helpful for surgeons and further research on otoplasty.

A careful analysis of the type and degree of auricular deformity is necessary to choose the best surgical technique. The choice also depends on the experience of the surgeon, who must obtain lasting and natural results. Both surgical and non-surgical approaches continue to require more research.

The eclectic technique with conchal remaining suture in the mastoid region proved to be effective in protruding ear correction. It provides anchoring precision in the pinna. Through fixation, retro positioning combined with superior rotation gives a natural and singular aesthetic aspect, with unnecessary incisions or cartilaginous interruptions and the consequent risk to the unwanted healing result. The low prevalence of complications allows us to disseminate this technique. Surgical revisions, if necessary, are limited and accurate, usually easy to perform.

## Conclusion

The alginate mold used to measure the cephalo-auricular and scapho-conchal angles in otoplasty was effective when comparing the two surgical techniques with and without preservation of the conchal cartilage. In the measurement of the angles and in the reduction of the distance after six months no significant differences were found. Both techniques fulfill their objectives, but one cannot assert the superiority over the other without carrying out more comparative studies.

## Compliance with ethical standards

All procedures performed in studies involving human participants were in accordance with the ethical standards of the institutional and/or national research committee and with the 1964 Helsinki declaration and its later amendments or comparable ethical standards.

## Conflicts of interest

The authors declare no conflicts of interest.

## Funding

This research has not received any specific funding from agencies of the public, commercial, or non-profit sectors.
